# Supplementary material for: Convolutional neural network-based classification of glaucoma using optic radiation tissue properties
Source: Commun Med (Lond). 2024 Apr 11;4:72. doi: 10.1038/s43856-024-00496-w (PMC11009254; doi:10.1038/s43856-024-00496-w)
Supplement: Supplementary file 2 — SUPPLEMENTAL MATERIAL [file 43856_2024_496_MOESM2_ESM.pdf]

Supplemental Material for “Convolutional neural network-based classification of glaucoma using optic radiation tissue properties”

John Kruper[1,2], Adam Richie-Halford[3], Noah C. Benson[2], Sendy Caffarra[3,4], Julia Owen[5,6], Yue Wu[5,6], Catherine Egan[7], Aaron Y. Lee[5,6], Cecilia S. Lee[5,6], Jason D. Yeatman[3], Ariel Rokem[1,2,*], and UK Biobank Eye and Vision Consortium[+]

[1]Department of Psychology, University of Washington, Seattle, WA, United States of America

[2]eScience Institute, University of Washington, WA, United States of America

[3]Graduate School of Education and Division of Developmental Behavioral Pediatrics, Stanford University, Stanford, CA, United States of America

[4]University of Modena and Reggio Emilia, Modena, Italy

[5]Department of Ophthalmology, University of Washington, Seattle, WA, United States of America

[6]Roger and Angie Karalis Johnson Retina Center, Seattle, WA, United States of America

[7]Moorfields Eye Hospital, NHS Trust, London, United Kingdom (C.E.)

[+]A list of authors and their affiliations appears at the end of the main text

[*]arokem@uw.edu

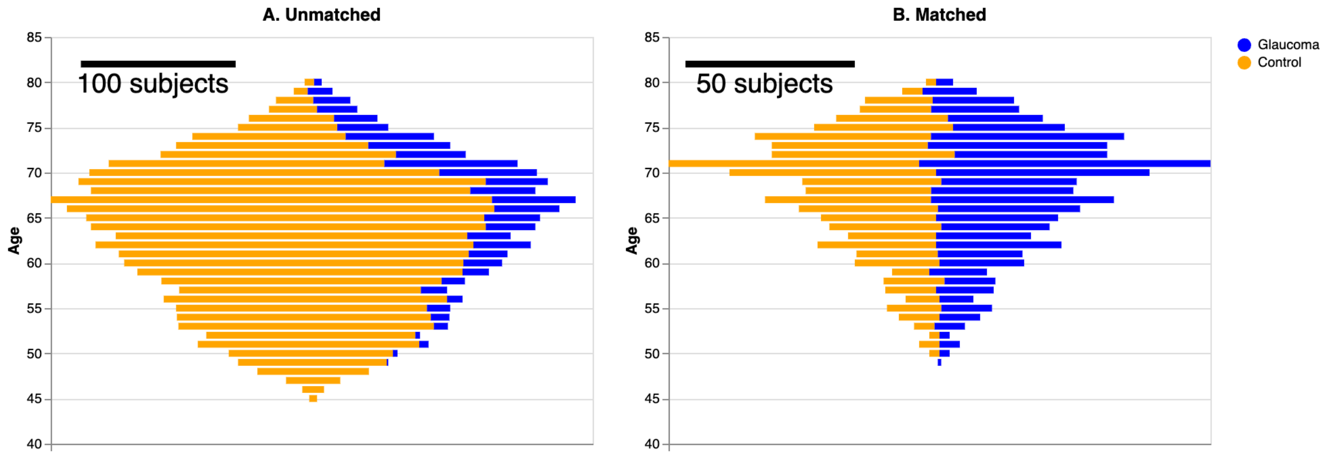

Supplementary Figure S1 Age distribution of subjects, colored by glaucoma status. The left panel shows the entire dataset before matching. There are significantly more controls (in orange) than subjects with glaucoma (scale bars are left: 100 subjects, right: 50 subjects). Additionally, the glaucoma subjects tend to be older. The right panel shows the dataset after matching each subject with glaucoma to a control subject with the same or similar age, sex, ethnicity, and the Townsend deprivation index (TDI)[?].

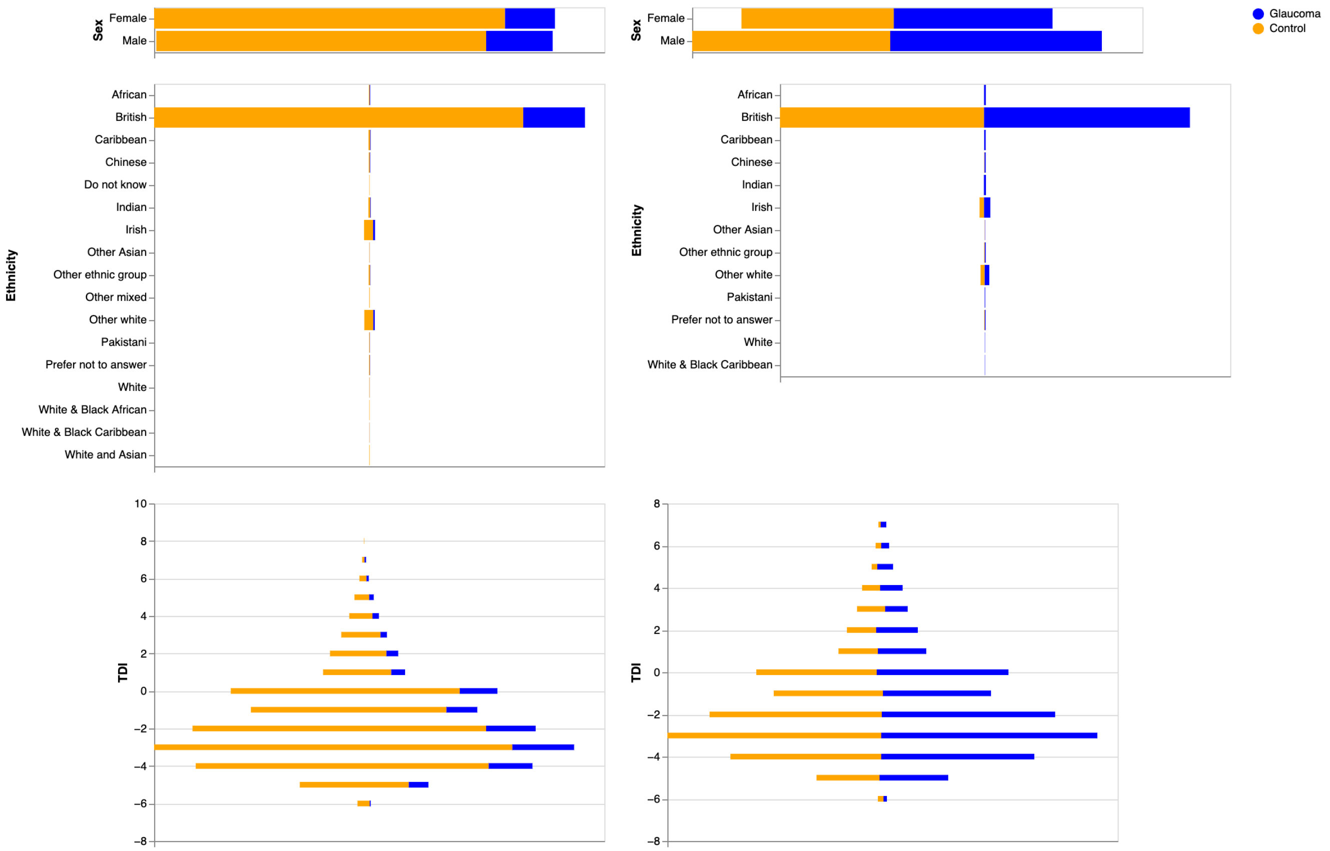

Supplementary Figure S2 Distributions of sex, ethnicity, and TDI of subjects, colored by glaucoma status. The left panels show the entire dataset before matching. The right panel shows the dataset after matching each subject with glaucoma to a control subject with the same or similar age, sex, ethnicity, and TDI.

Supplementary Table S1 Confusion matrix for CNN trained on OR (top) and logistic regression trained on OR (bottom) for glaucoma classification, with classification threshold set to 0.5

	CNN Predicted: Control	CNN Predicted: Glaucoma
**Actual: Control**	112 (32.56%)	60 (17.44%)
**Actual: Glaucoma**	64 (18.60%)	108 (31.40%)

	Reg. Predicted: Control	Reg. Predicted: Glaucoma
**Actual: Control**	105 (30.52%)	67 (19.48%)
**Actual: Glaucoma**	69 (20.06%)	103 (29.94%)

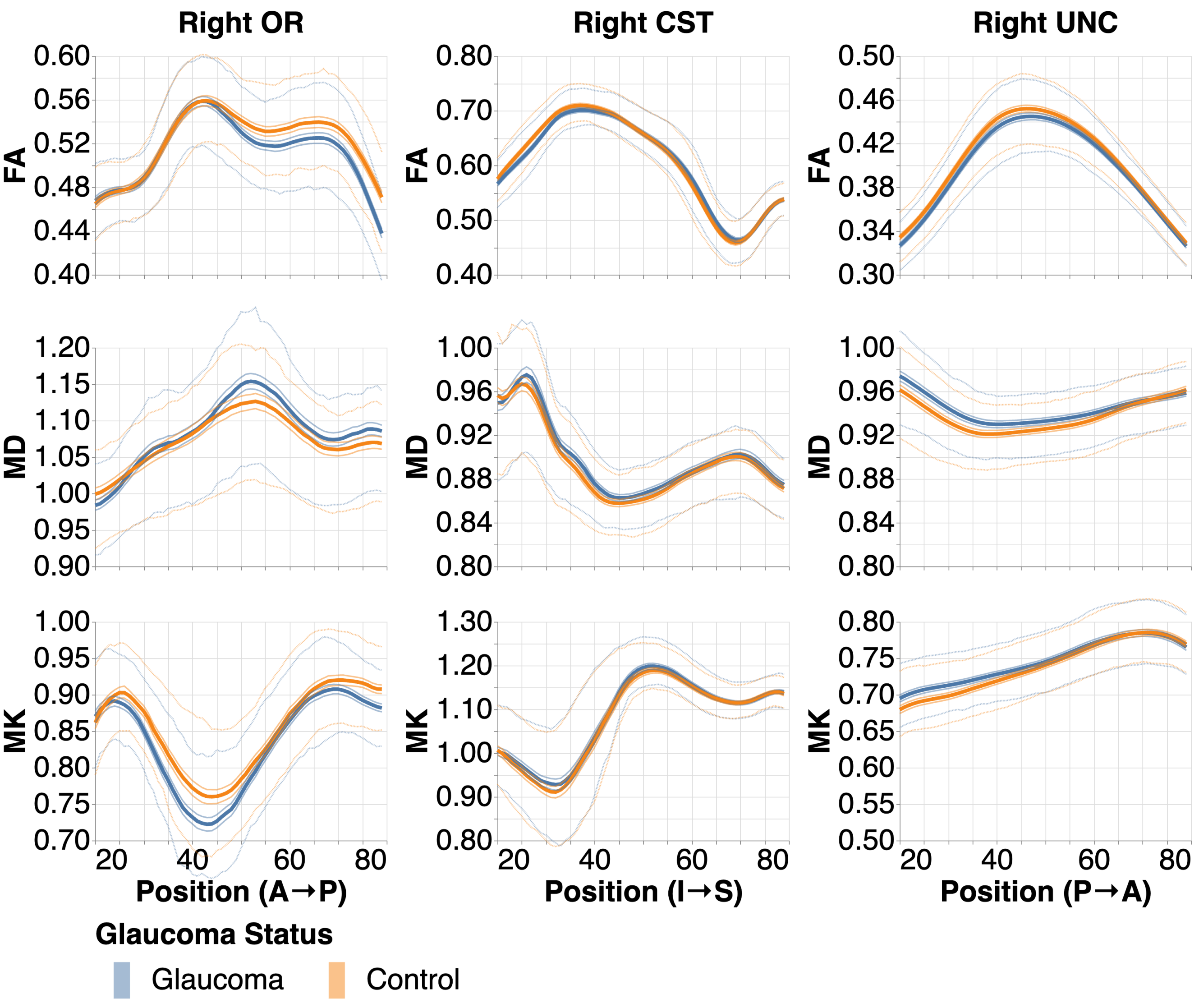

Supplementary Figure S3 Thick lines show the mean tract profiles in the right hemisphere of all bundle and tissue property combinations. The medium-thickness lines hugging the thick lines show the 95% confidence interval. The thin lines show interquartile ranges. Positions in OR are from anterior to posterior (A→P), in the corticospinal tract (CST) are from inferior to superior (I→S), and in the uncinate (UNC) from posterior to anterior (P→A).

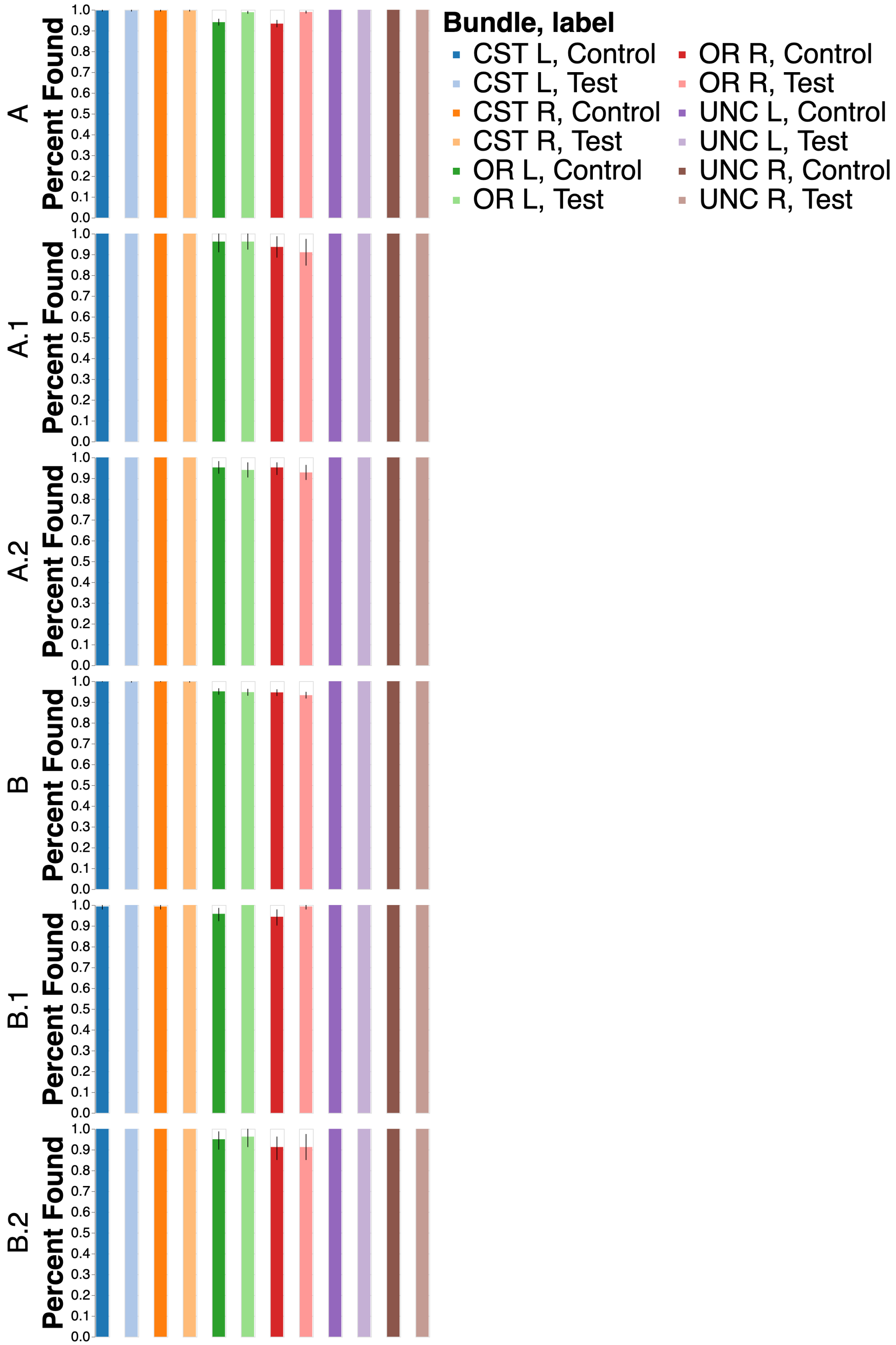

Supplementary Figure S4 Percentage of successfully delineated bundles in each dataset, separated by classification label. The OR sub-bundles are harder to track than the controls, and are sometimes not found. Uncertainties show a bootstrapped 95% confidence interval. Note that, in dataset A, the control subjects are not as likely to have successfully delineated OR as the glaucoma subjects, but see control analysis in Supplementary Figure S5.

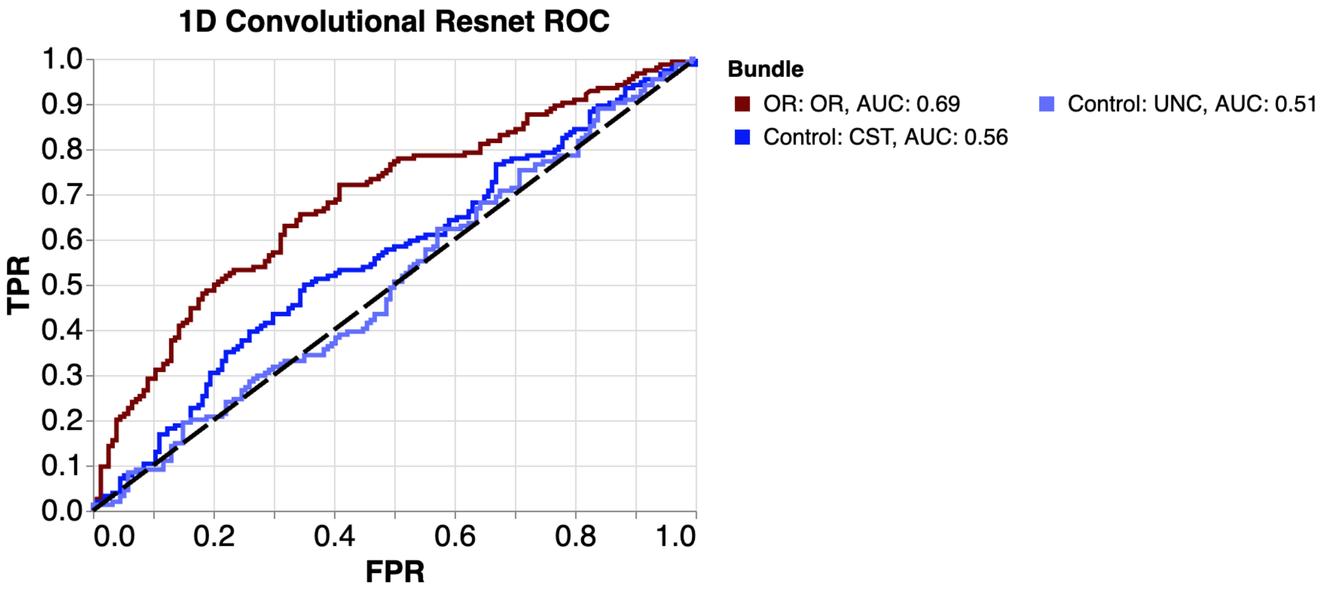

Supplementary Figure S5 ROC curves for prediction of glaucoma using the three neural networks trained on dataset A. Only subjects with no missing bundles are included in the test dataset used to make these ROC curves. This reduces the number of matched pairs of test subjects from 172 to 154 (308 subjects in total). As in Figure 3, the OR have statistically significantly higher AUC than the control bundles (CST p=0.0043, UNC p=0.0001).
