# Supplementary material for: Convolutional neural network-based classification of glaucoma using optic radiation tissue properties
Source: Commun Med (Lond). 2024 Apr 11;4:72. doi: 10.1038/s43856-024-00496-w (PMC11009254; doi:10.1038/s43856-024-00496-w)
Supplement: Supplementary file 3 — Reporting Summary [file 43856_2024_496_MOESM3_ESM.pdf]

nature portfolio

Corresponding author(s): Ariel Rokem

Last updated by author(s): Sep 19, 2023

Reporting Summary

Nature Portfolio wishes to improve the reproducibility of the work that we publish. This form provides structure for consistency and transparency in reporting. For further information on Nature Portfolio policies, see our Editorial Policies and the Editorial Policy Checklist.

Statistics

For all statistical analyses, confirm that the following items are present in the figure legend, table legend, main text, or Methods section.

n/a	Confirmed	
☐	☒	The exact sample size (*n*) for each experimental group/condition, given as a discrete number and unit of measurement
☐	☒	A statement on whether measurements were taken from distinct samples or whether the same sample was measured repeatedly
☐	☒	The statistical test(s) used AND whether they are one- or two-sided<br>*Only common tests should be described solely by name; describe more complex techniques in the Methods section.*
☐	☒	A description of all covariates tested
☐	☒	A description of any assumptions or corrections, such as tests of normality and adjustment for multiple comparisons
☐	☒	A full description of the statistical parameters including central tendency (e.g. means) or other basic estimates (e.g. regression coefficient) AND variation (e.g. standard deviation) or associated estimates of uncertainty (e.g. confidence intervals)
☐	☒	For null hypothesis testing, the test statistic (e.g. F, t, r) with confidence intervals, effect sizes, degrees of freedom and P value noted<br>*Give P values as exact values whenever suitable.*
☒	☐	For Bayesian analysis, information on the choice of priors and Markov chain Monte Carlo settings
☒	☐	For hierarchical and complex designs, identification of the appropriate level for tests and full reporting of outcomes
☐	☒	Estimates of effect sizes (e.g. Cohen's *d*, Pearson's *r*), indicating how they were calculated

Our web collection on statistics for biologists contains articles on many of the points above.

Software and code

Policy information about availability of computer code

Data collection	This study uses publicly available data from the UK Biobank.
Data analysis	All code to reproduce the analysis and the figures is available at: https://github.com/36000/glauc_paper_scripts<br>This github includes versions for all software needed to reproduce the research.

For manuscripts utilizing custom algorithms or software that are central to the research but not yet described in published literature, software must be made available to editors and reviewers. We strongly encourage code deposition in a community repository (e.g. GitHub). See the Nature Portfolio guidelines for submitting code & software for further information.

Data

Policy information about availability of data

All manuscripts must include a data availability statement. This statement should provide the following information, where applicable:

- Accession codes, unique identifiers, or web links for publicly available datasets
- A description of any restrictions on data availability
- For clinical datasets or third party data, please ensure that the statement adheres to our policy

This study uses publicly available data from the UK Biobank. More information on the data and access can be found here: https://www.ukbiobank.ac.uk/enable-your-research

Human research participants

Policy information about studies involving human research participants and Sex and Gender in Research.

Reporting on sex and gender	We only use the term sex and our findings only apply to sex. We used data field 31 from the UK Biobank, described as: " Acquired from central registry at recruitment, but in some cases updated by the participant. Hence this field may contain a mixture of the sex the NHS had recorded for the participant and self-reported sex. "
Population characteristics	Our participants tend to be aging and ethnically British. For specifics, see Supplementary Figures 1 & 2. Our test population has glaucoma.
Recruitment	We selected from UK Biobank participants where diffusion MRI data was collected. From the available data, we created several distinct datasets, using statistical matching to create datasets where bias due to age and other factors is negligible.
Ethics oversight	UK Biobank Eye and Vision Consortium

Note that full information on the approval of the study protocol must also be provided in the manuscript.

Field-specific reporting

Please select the one below that is the best fit for your research. If you are not sure, read the appropriate sections before making your selection.

☒ Life sciences ☐ Behavioural & social sciences ☐ Ecological, evolutionary & environmental sciences

For a reference copy of the document with all sections, see nature.com/documents/nr-reporting-summary-flat.pdf

Life sciences study design

All studies must disclose on these points even when the disclosure is negative.

Sample size	Our sample size was determined by the number of subjects in the UK Biobank who fit the criteria for a given dataset. Datasets are described in the methods section of the paper. Our main dataset has 856 glaucoma subjects and 856 matched control subjects. These sample sizes are an order of magnitude larger than any previous study using diffusion MRI to look at glaucoma.
Data exclusions	We also only include subjects with a final visual acuity logMAR of less than or equal to 0.3 for the results shown, but we re-ran the analyses without this criterion and found the same results. We do this such that subjects with extreme differences in vision are not driving the main effect.
Replication	We re-ran the experiment several times while changing some of the parameters: the covariates in the statistical matching, the statistical matching criterion, exclusion criterion, and neural network architecture used. In all cases, the results replicate.
Randomization	Subjects were divided into experimental groups by either age, glaucoma status, or age related macular degeneration status, depending on the dataset. Covariates were controlled two ways. One, we used statistical matching to reduce the effects of age, sex, and ethnicity. Two, we compared effects in visual bundles to non-visual bundles, both of which would be affected by most covariates. Still, the limitations of this setup are discussed in the discussion.
Blinding	Blinding was not possible as we used an already collected dataset. The limitations of this approach are discussed in the discussion.

Reporting for specific materials, systems and methods

We require information from authors about some types of materials, experimental systems and methods used in many studies. Here, indicate whether each material, system or method listed is relevant to your study. If you are not sure if a list item applies to your research, read the appropriate section before selecting a response.

Materials & experimental systems

n/a	Involved in the study
☒	☐ Antibodies
☒	☐ Eukaryotic cell lines
☒	☐ Palaeontology and archaeology
☒	☐ Animals and other organisms
☒	☐ Clinical data
☒	☐ Dual use research of concern

Methods

n/a	Involved in the study
☒	☐ ChIP-seq
☒	☐ Flow cytometry
☒	☐ MRI-based neuroimaging
